# Experimental Investigation of Fatigue Debonding Growth in FRP–Concrete Interface

**DOI:** 10.3390/ma13061459

**Published:** 2020-03-23

**Authors:** Xinzhe Min, Jiwen Zhang, Chao Wang, Shoutan Song, Dong Yang

**Affiliations:** 1 School of Civil Engineering, Southeast University, Nanjing 210096, China; xzminseu@foxmail.com (X.M.); chao.wang.seu@gmail.com (C.W.); songshoutan@seu.edu.cn (S.S.); dongyang@seu.edu.cn (D.Y.); 2 Key Laboratory of Concrete and Prestressed Concrete Structures of the Ministry of Education, Southeast University, Nanjing 210096, China

**Keywords:** carbon fiber reinforced polymer (CFRP), adhesion, interface, pull-out test, fatigue, debonding growth

## Abstract

An externally bonded fiber reinforced polymer (FRP) plate (or sheet) is now widely used in strengthening bending members due to its outstanding properties, such as a high strength to weight ratio, easy operating, corrosion and fatigue resistance. However, the concrete member strengthened by this technology may have a problem with the adhesion between FRP and concrete. This kind of debonding failure can be broadly classified into two modes: (a) plate end debonding at or near the plate end, and (b) intermediate crack-induced debonding (intermediate crack-induced (IC) debonding) near the loading point. The IC debonding, unlike the plate end debonding, still needs a large amount of investigation work, especially for the interface under fatigue load. In this paper, ten single shear pull-out tests were carried out under a static or fatigue load. Different load ranges and load levels were considered, and the debonding growth process was carefully recorded. The experimental results indicate that the load range is one of the main parameters, which determines the debonding growth rate. Moreover, the load level can also play an important role when loaded with the same load range. Finally, a new prediction model of the fatigue debonding growth rate was proposed, and has an excellent agreement with the experimental results.

## 1. Introduction

During these few decades, the carbon fiber reinforced polymer (CFRP) stood out from many strengthening materials for its numerous advantages, such as a high strength to weight ratio, corrosion and fatigue resistance [1,2,3,4,5]. At present, the most popular way to practically apply this material in flexural strengthening is to externally bond the FRP plate (or sheet) on the tension face of the bending member. This method has been proofed to be a highly efficient method in increasing the load capacity, decreasing the mid-span deflection, delaying the crack development, etc. [6,7,8,9,10,11]. 

Based on previous research, by using of externally bonded FRP plate, several premature debonding failures [12,13,14,15,16,17,18,19] may occur before the load reaching the expected failure modes, such as a rupture of the FRP plate or concrete crushing, and thus lead to unsatisfactory strengthening effects. The failure modes caused by FRP debonding can be broadly classified into two categories [20]: (a) plate end debonding: separation of the FRP plate or FRP plate attached with the whole thickness of the concrete cover from (or near) the plate end and then propagating towards the loading point; and (b) intermediate crack-induced debonding (IC debonding): debonding caused by the opening of the major flexural crack or flexural–shear crack and propagating towards the near plate end. A lot of strength models for plate end debonding have been proposes by a large amount of studies [21,22,23]. The plate end debonding can also be prevented by using of plate end anchorages [24,25]. However, applying the externally bonded FRP strengthening on slender bending members, such as the girders of a bridge, IC debonding has become more commonly observed and worthy concerning [26,27,28]. The main reason is that, as IC debonding occurred near the major flexural crack (or flexural–shear crack), the tension force originally carried by the FRP plate will redistribute into the steel reinforcement and increase its stress, which would lead to many problems such as an unexpected shorter fatigue life of a strengthened member. To observe and study IC debonding in beam tests is extremely hard due to the uncertainty of the location of major cracks. Therefore, a single (or double) shear pull-out test, which has the same mechanism as the vicinity of IC debonding, has been adopted by many researchers [29,30,31].

At present, a large amount of studies have been concentrating on the FRP–concrete interface under static load [32,33], but the fatigue behavior of the interface still need more researches. Bizindavyi et al. [34] conducted tests of the FRP–concrete interface considering various parameters, such as: different FRP materials (glass fiber reinforced polymer, GFRP, and carbon fiber reinforced polymer, CFRP), different FRP thickness or width, different load amplitudes and mean levels, different number of FRP layers and different bond length or width. The study showed that a higher load range could lead to a shorter fatigue life, as well as a shorter bond length and higher stress level. Additionally, *P*_min_ (lower limit of fatigue load) influenced the fatigue life even the interfaces were loaded under the same load range. Bond joints loaded with *P*_min_ = 0 had a greater fatigue life than those with *P*_min_ ≠ 0. Finally, an S-N (stress level - fatigue life) curve was proposed. Dial et al. [35] presented an analytical solution of the fatigue debonding growth of the FRP–concrete interface under mode-II fatigue loading, and the corresponding pull-out tests were also conducted. The upper limit of fatigue loading varied from 30% to 80% of the load capacity obtained from static tests. The authors provided a threshold value of 50% of the static bond capacity for preventing the fatigue debonding failure, and 30% of the static bond capacity for eliminating debonding. The proposed prediction model of the debonding growth rate *da/dN* (the length of debonding growth per cycle) suggested that *da/dN* can be determined by the fracture energy ratio, the fatigue load frequency and the debond length that has already developed. Carloni et al. [36] applied DIC (digital image correlation) on analyzing the strain distribution on FRP under a static and fatigue load. The slopes of the hysteresis loops of the bond joint were gradually decreasing with the number of cycles, which indicated the loss of interfacial stiffness. Moreover, the shorter stress transfer zone, for stress transfer from FRP to concrete, was found after fatigue load application. An improvement of Diab’s [35] prediction model was proposed, but the parameters were left undetermined due to the lack of experimental data. Daud et al. [37] tested FRP–concrete interface with different FRP stiffness under fatigue loading. The load amplitude has a significant effect on fatigue life of the bond joint. The results indicated that FRP with lower stiffness has higher debonding strain and the debonding strain would also reduce after prior fatigue load was applied. In this paper, behaviors of the FRP–concrete interface under static and fatigue load were investigated. Parameters including a fatigue load range and fatigue load level were considered. The load–slip response and strain distribution on the FRP plate were recorded at certain check cycles. To study the debonding propagation, strain profile analysis after tests and direct observation during the tests were adopted. The results indicate the significant influence of load level when the load range was the same. Finally, a new prediction model was proposed to reveal the relationship between fatigue debonding growth along the FRP–concrete interface and the load range and load level.

## 2. Research Significance

The main objectives of this study are:

(1) To study the load capacity, the stress transfer length and failure mode of the FRP–concrete interface under a monotonic load;
(2) To investigate the behavior and failure mode of the FRP–concrete interface under fatigue loads with different fatigue load ranges and load levels;
(3) To define the rate of the debonding growth along the FRP–concrete interface under fatigue loads with different loading parameters;
(4) To propose a prediction model, which can correctly describe this fatigue debonding growth rate.

## 3. Materials and Methods

Ten single shear pull-out tests were conducted under monotonic or fatigue loadings. Specimens were made of concrete substrate bonded with unidirectional CFRP plate. The FRP concrete interface was loaded statically to failure to define the load capacity, in order to determine the following fatigue loadings. The fatigue tests consisted of groups with different load ranges and load levels. The upper limits of the fatigue load ranged from 60% to 80% of the ultimate load obtained from static tests and the lower limits varied as well. All fatigue loadings were at a frequency of 5 Hz. Experimental program is summarized in Table 1.

In Table 1, the lower and upper limits of the fatigue loads are given in a normalized form, same with the load range and load level. *S*_min_ = *P*_min_/*P*_u_ is the relative lower limit of fatigue load; *S*_max_ = *P*_max_/*P*_u_ is the relative upper limit of fatigue load; *ΔS* = (*S*_max_ − *S*_min_ )/2 and $\bar{S}$ = (*S*_max_ + *S*_min_ )/2 are the relative load range and relative load level of the fatigue load; *P*_min_ and *P*_max_ are the lower and upper limit of applied fatigue load, respectively; *P*_u_ is the load capacity of the FRP–concrete interface obtained from static tests. From FT-1 to FT-5, the upper limits of the fatigue tests vary from 0.6 to 0.8 *P*_u_, and the lower limits stay the same. FT-6 shares the same load range with FT-1, but higher load level. FT-7 and FT-4 also have the same load range but different load levels. FT-6 and FT-4 have the same upper limit of the fatigue load but FT-6 has higher lower limit.

### 3.1. Details of Test Specimens

**Figure 1:**
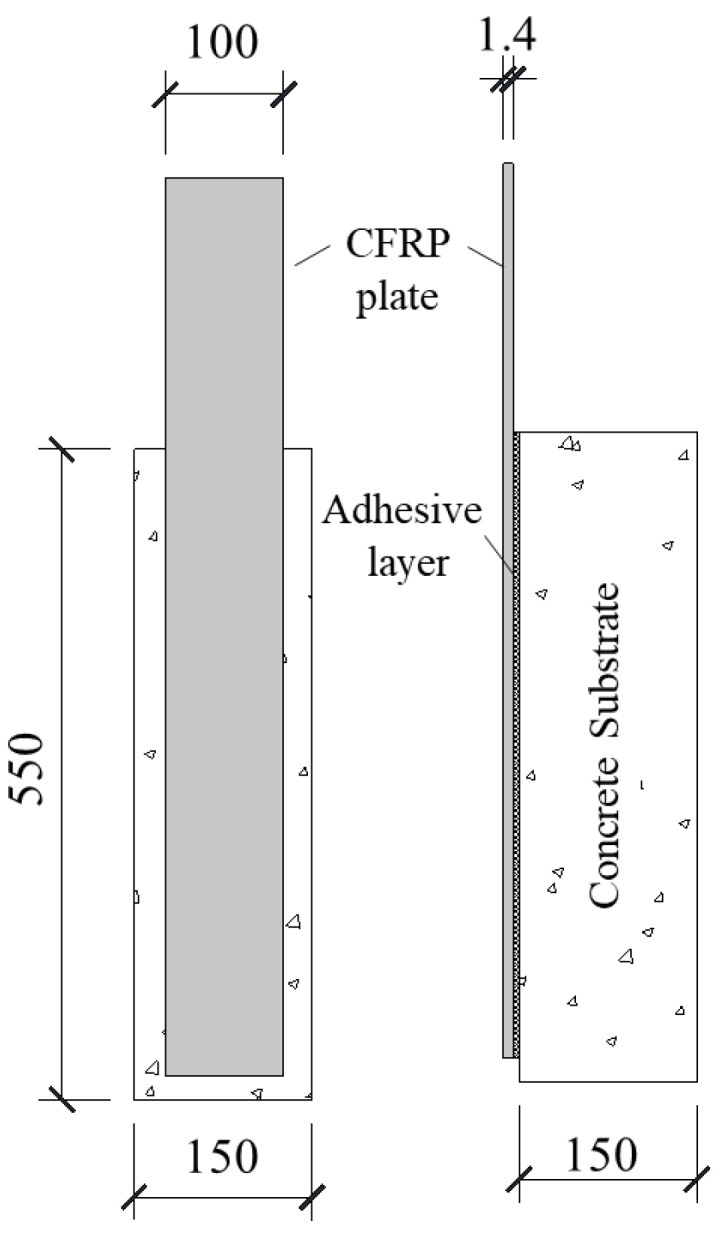
Loading configuration and specimen.

The details of the tested specimen are shown in Figure 1. The nominal cross section dimensions of the CFRP plates was 1.4 mm thick by 100 mm wide. The length of the CFRP plates was 1000 mm and the bond length was 500 mm, which was much longer than the effective bond length [38]. The Young’s modulus and tensile strength of the unidirectional CFRP plate were 2.45 GPa and 170 GPa [39], respectively. The dimensions of the concrete blocks were 550 mm in length, 150 mm in both width and thickness. Commercial concrete was used in this study and the mix proportion of which is cement: fine aggregate: coarse aggregate: water = 1:1.07:2.08:0.43. The average measured cubic compressive strength of the four tested cubic concrete blocks was 31 N/mm^2^ [40].

### 3.2. Surface Preparation and Bond Process

**Figure 2:**
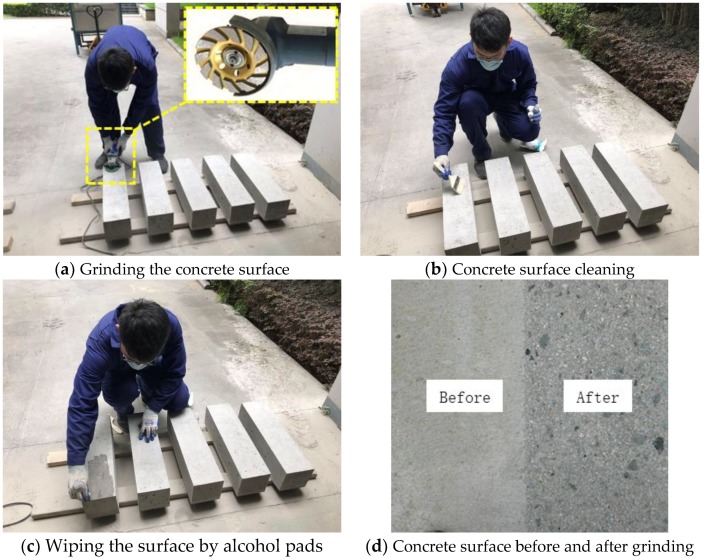
Concrete surface preparation.

The surface preparation of concrete substrate consisted of two major steps, which were grinding and cleaning. First, the surface was grinded to certain depth by an abrasive dry grinder to expose most of the aggregates. This step was intended to remove the laitance on the top of concrete substrate, which would result in weak bonding between CFRP and concrete. Secondly, the grinded surface was cleaned by a brush and vacuum cleaner and wiped several times by alcohol pads to remove dust and particles. The photos of the surface preparation process and the comparison of the concrete surface before and after grinding were shown in Figure 2.

The adhesive used in this work was provided by Nanjing Hitech Composites Co. Ltd. (Nanjing, China) and the type of the adhesive was Lica-131 A/B. The bond process was carried out after the concrete surface becoming totally dried. The mixture proportions by weight of the epoxy resin adhesive were epoxy resin: epoxy hardener = 2:1. The physical properties of the adhesive were listed in Table 2. The adhesive was placed around the longitudinal center line on the CFRP plate, while attaching to the concrete substrate, the CFRP plate was pressed to exclude air along the interface. Considering that the 1.4 mm thick CFRP plate is quite firm, few glass balls with a 2 mm diameter were scattered on the adhesive layer before bonding. Then, by pressing the CFRP plate against the concrete, the redundant adhesive was squeezed out, and the thickness of the adhesive layer would be controlled around 2 mm.

### 3.3. Test Set-Up

**Figure 3:**
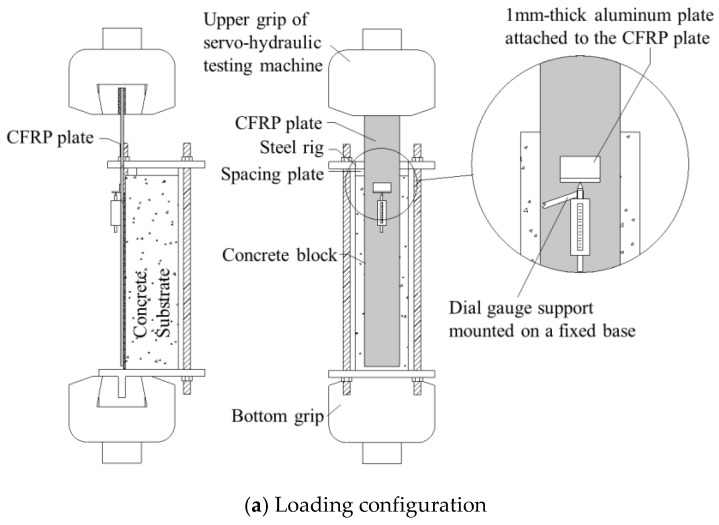

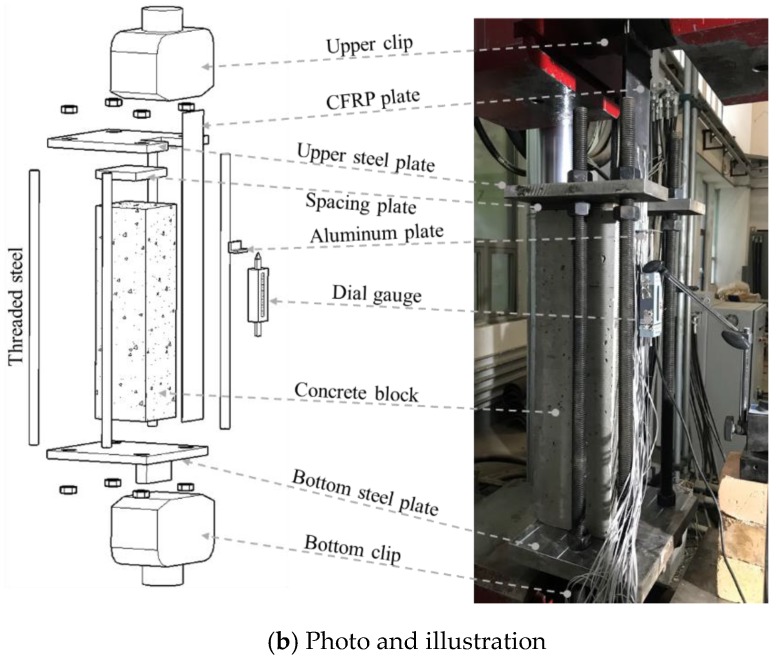
Single shear pull-out test configuration.

All the tests were performed under a typical single shear pull-out test configuration as shown in Figure 3. The concrete block was constrained by a steel rig, which consisted of two 30 mm thick steel plates and four 24 mm diameter high-strength threaded steels. Both steel plates had four holes at four corners to let the threaded steels pass through and fixed by nuts. For the bottom plate, another 20 mm thick steel plate (gripping plate) was perpendicularly welded on the underside, which could be firmly clamped by the lower hydraulic clip of the servo-hydraulic testing machine. This steel plate was placed along the longitudinal axis of the CFRP plate. This arrangement was designed to ensure that the whole testing system could be axially loaded and excessive trembling could be prevented under fatigue loading.

The CFRP plate was clamped by the upper hydraulic clip. When loaded, stress would gradually transfer from the CFRP plate to the concrete substrate through the FRP–concrete interface. The specimens (CFRP attached concrete blocks) were carefully placed before testing to ensure the upper clip, the CFRP plate and the gripping plate were along the same axis, so that the loaded system could be steady during static and fatigue tests. In addition, a 20 mm thick steel plate was inserted between the top of the concrete block and the steel rig, so that shear crack could develop in the concrete block.

### 3.4. Instrumentation and Testing Procedure

**Figure 4:**
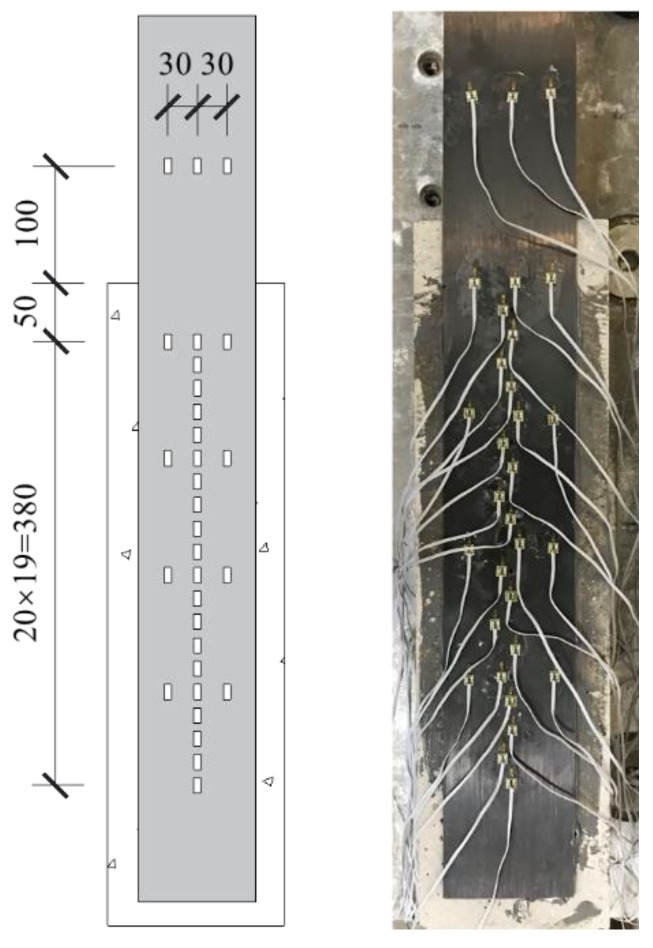
Detail of strain gauges arrangement.

Longitudinal strain distribution of the CFRP plate was measured by densely installed strain gauges. Thirty-one strain gauges were mounted along the CFRP fiber direction, twenty-one of which were installed along the central axis and ten were transversely mounted to study the stain distribution along the plate width direction. The detail of strain gauges arrangement was shown in Figure 4.

The relative slip between FRP and the concrete block was measured by the dial gauge. In this study, the relative slip was defined as the displacement between the aluminum plate (attached on the FRP plate along the upper edge of the bond region) and a fixed base as shown in Figure 3. The measured data of strain distribution and relative slip were recorded by the data logger (TDS530) continually at a frequency of 1 Hz.

## 4. Results and Discussions

### 4.1. Monotonic Tests

Three specimens MT-1, MT-2 and MT-3 were tested under monotonic loading up to failure. All the specimens were loaded at a constant rate equal to 0.005 mm/s (stroke rate of the upper clip of the servo-hydraulic testing machine) until the failure occurred. Relative slip between the concrete substrate and the CFRP plate was measured throughout each test. 

#### 4.1.1. Failure Mode

**Figure 5:**
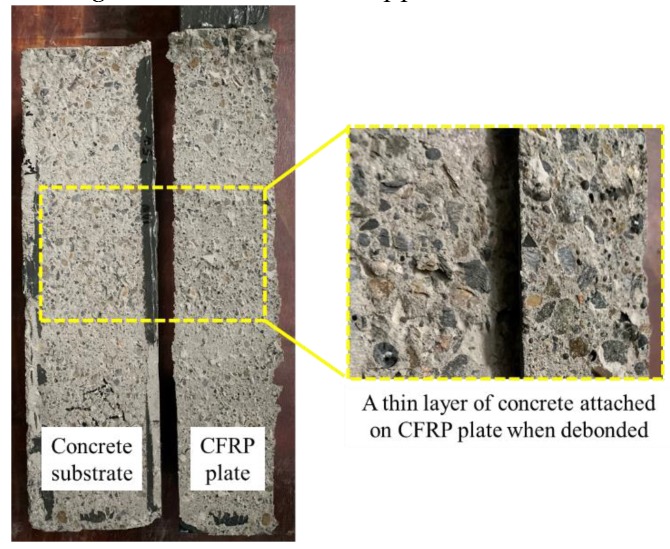
Failure mode of the fiber reinforced polymer (FRP)–concrete joint under static load.

All the FRP–concrete joints expressed the same failure mode: debonding of the CFRP plate with a few millimeters thick of concrete attached on it, as shown in Figure 5. The measured ultimate loads, *P*_u_, of the joints were listed in Table 3. The average load capacity of the FRP–concrete interface was 45 kN, which was used in fatigue tests to define the upper and lower limits for fatigue load.

#### 4.1.2. Load–Slip Relationship

**Figure 6:**
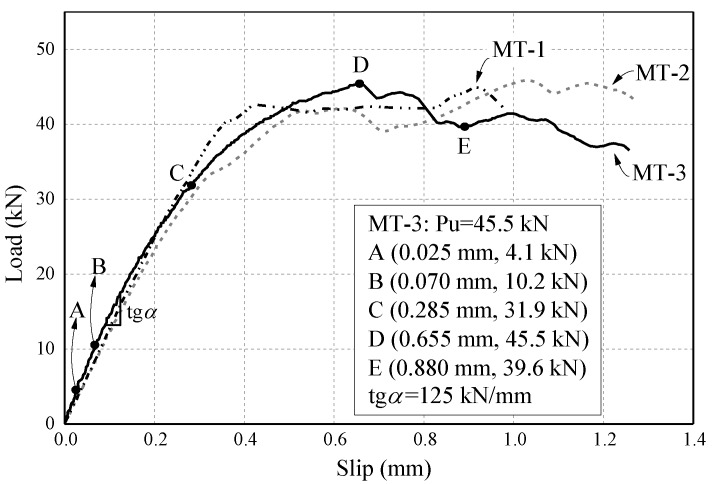
Load–slip relationship of the monotonic pull-out tests.

Figure 6 presents the load–slip relationships of all the monotonic pull-out tests. The general behavior of the FRP–concrete interface can be described by the applied load–relative slip (load–slip) relationship. Typically, MT-3 as an example, a load–slip curve of an FRP–concrete interface started with a linear increasing potion, then a non-linear relationship would show up before the curve reaching the ultimate load. After this peak point, the load–slip curve attended to level off. With the slip increasing, a visible crack growth can be found along the longitudinal direction of the joint. Finally, the monotonic test ended by a sudden drop of the applied load, which was also related to an explosive rupture of the remaining last part of the bond joint. 

It is worthy to mention that, the ultimate load of the bond joint may not be reached at the first peak point, in another word, the applied load could still increase after a short decreasing portion of the load–slip curve. This is because the quality of the bond joint is inconsistent along the entire length of the joint. Furthermore, load capacity of the FRP–concrete interface mainly depends on the bond quality within the effective bond length [11,13,16]. So, if the bond joint is long enough, as debonding propagating, different parts of the joint with slightly difference in bond quality would be tested, an ultimate load can be reached within any segment of the joint.

#### 4.1.3. FRP Strain Distribution

**Figure 7:**
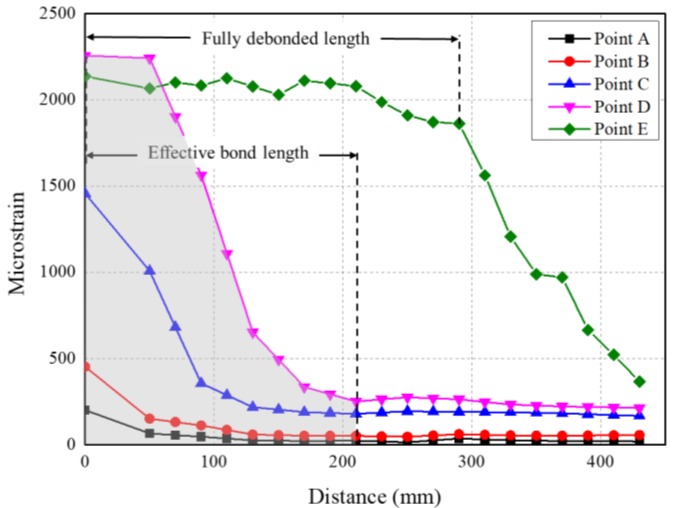
FRP strain distribution of MT-3 at each load point.

As shown in Figure 6, MT-3 as an example, five points were marked on the load–slip relationship curve during different loading phases, which were the low-stress phase (Point A), linear response phase (Point B), non-linear response phase (Point C), peak point (Point D) and debonding growth phase (Point E). The strain distribution on the CFRP plate at each point was presented in Figure 7 respectively. 

The strain distribution curve at points before the peak load can be divided into two segments: the stress transfer segment with a high strain gradient and the low stress segment, which was almost parallel to the X-axle. Tensile stress on the CFRP plate gradually transfers to the concrete substrate through the interface. With the increasing load applied to the CFRP plate, the length of this portion extended and strain kept rising until the ultimate load was reached. At the peak point (Point D), the stress transfer length (210 mm) stayed constant and equaled the effective bond length referring to Chen and Teng strength model [38]. After the peak load, the general shape of the curve within the stress transfer length generally kept constant and moved towards the free end of the bonded CFRP plate with the increasing slip. Meanwhile another almost horizontal segment, fully debonded segment, gradually formed from the loaded end. This high-strain level segment implied the separation between the CFRP plate and concrete. Debonding growth can also be observed by direct observation. Additionally, it matched well with the strain profile.

### 4.2. Fatigue Tests

Seven single shear pull-out tests were conducted under constant amplitude fatigue loading. These tests were designed to investigate the debonding propagation along the FRP–concrete interface under fatigue loading. In order to record relatively accurate debond length, attentive visual check during the test and strain profile analysis after the test were used and mutually proofread.

#### 4.2.1. Fatigue Loading Process and Data Recording

Cyclic loading was performed between a maximum load (*P*_max_) and a minimum load (*P*_min_), where *P*_max_ and *P*_min_ corresponded to certain percentages of the average ultimate load (*P*_u_) obtained from the monotonic tests. In a typical fatigue test, the specimen was loaded to the mean value of the cyclic load range at a rate of 0.05 kN/s, then the cyclic load was performed at 5 Hz until a certain cyclic number was reached or failure occurred.

**Figure 8:**
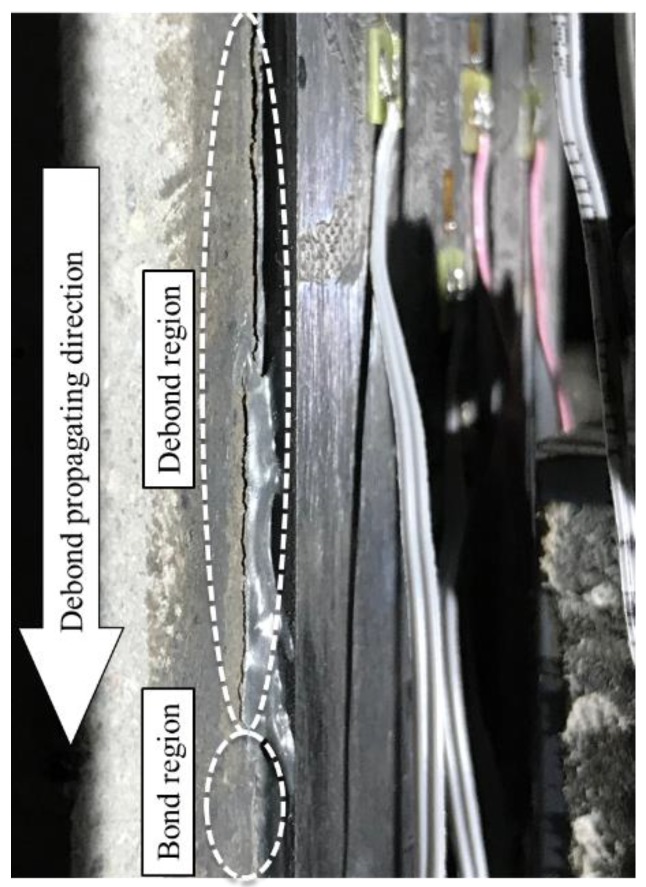
Debond propagation under fatigue load.

The fatigue tested specimens were loaded statically at some specific check cycles intended to test the load–slip response, strain profile and debonding propagating situation after certain amount of cyclic loading. The load was increasingly applied at a rate of 0.05 kN/s from *P*_min_ to *P*_max_ and decreased back to *P*_min_. During each round of static test, the end of debond area was carefully checked by visual observation, as shown in Figure 8, and marked on the concrete substrate by a marker pen, then measured the debond length. Combined with the analysis of CFRP plate strain profile, which would be described below, the actual debond length could be measured relatively accurately.

#### 4.2.2. Failure Mode and Fatigue Life

During cyclic loading, slight cracking sound could always be heard throughout the process. Visible reciprocating sliding of debonded CFRP plate, with a thin layer of concrete attached on it, along the loading direction can also be noticed. Dust and small particles generated from the friction between the debonded FRP plate and concrete substrate accumulated on the bottom steel plate below the FRP free end.

All the fatigue-loaded specimens had the same failure mode: progressive interfacial crack (debonding) propagation between the CFRP plate and concrete substrate up to a sudden explosive separation of the last bond joint, which was too short to carry the applied load. The results of all the tests were reported in Table 3.

#### 4.2.3. Load Response

**Figure 9:**
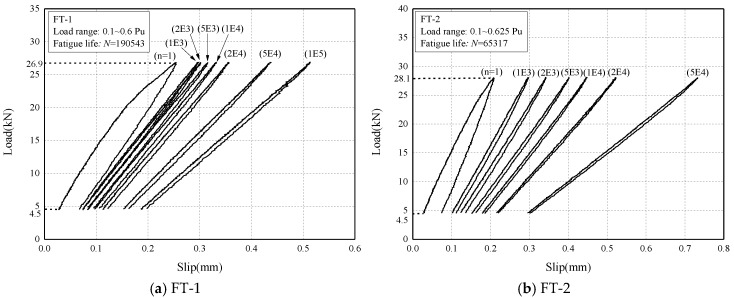

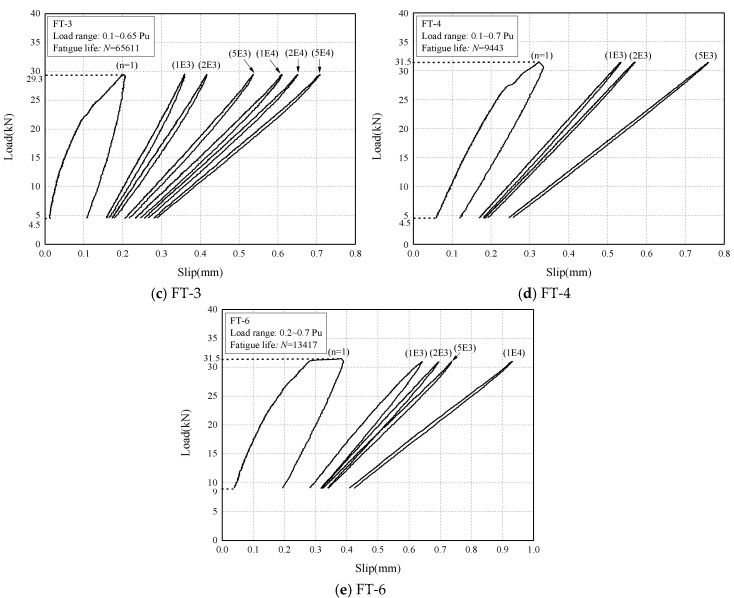
Load responses under fatigue loading (the number above each curve stand for corresponding cyclic number).

In Figure 9, load responses at different cycles of specimens with *N_f_* > 1000 were plotted. The decreasing in the slope of load–slip response was noticeable with the number of cycles, indicating the reduction in the bond stiffness between CFRP plate and concrete. The bond stiffness was the overall behavior of the bond joint, reduction in which implied greater deformation of unconstrained CFRP plate generated, in other words, debonding has already formed in the joint.

**Figure 10:**
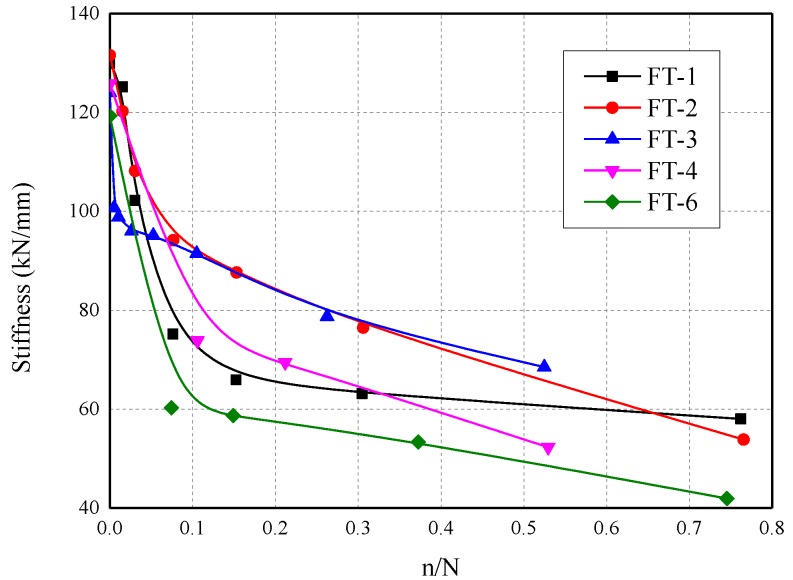
Secant bond stiffness in normalized fatigue cycles.

The secant bond stiffness corresponding to normalized fatigue cycles was plotted in Figure 10. Sharp reduction in the bond stiffness was observed in the initial cycles, as cycles increasing, the decreasing rate of bond stiffness slowed down. This phenomenon could be attributed to the friction between the debonded CFRP plate with the same rough surface of the opposite concrete substrate. The change in the decreasing rate of bond stiffness could be potentially explained by a larger debonded area that was involved into friction as interfacial damage accumulated.

It is worth noticing that the bond stiffness mentioned above was the overall performance of the bond joint. The slip between the CFRP plate and concrete substrate at the aluminum plate level consisted of tensile deformation of the debonded CFRP plate and the remaining bond joint, hence the actual bond stiffness of the joint was higher than the stiffness calculated in the tests. However, the bond stiffness obtained by the slope of the load–slip curve was much easier to get and has more extensive practical usage.

#### 4.2.4. Strain Distribution

**Figure 11:**
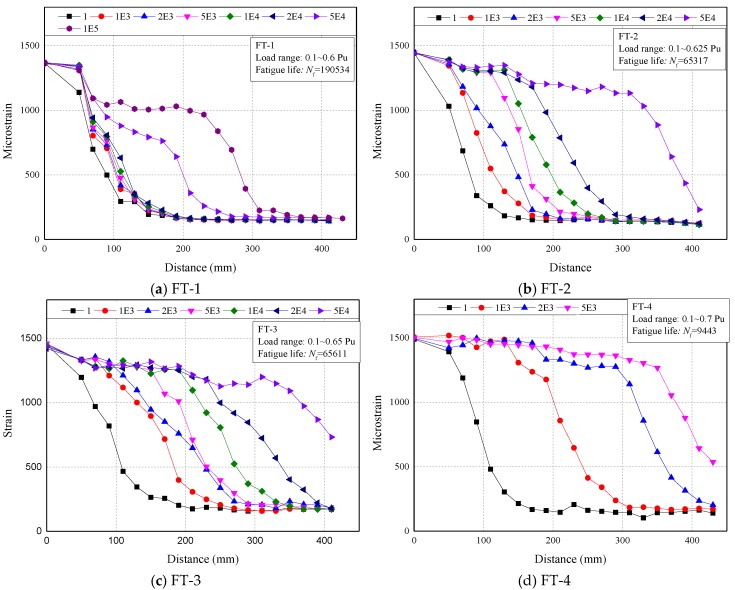

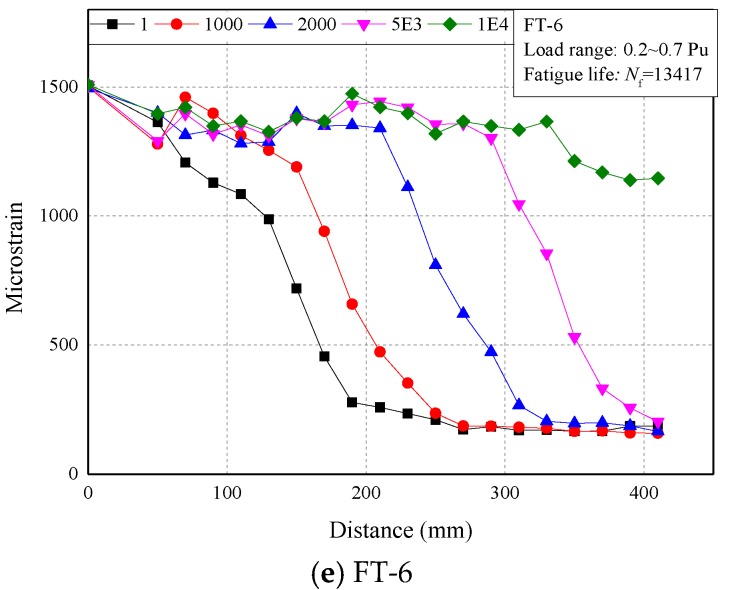
FRP strain profile at different fatigue load cycles.

In Figure 11, the longitudinal strain distributions on the CFRP plate of specimens with *N_f_* > 1000 under *P*_max_ at different loading cycles were presented. The evolution of the strain profile with increasing cyclic number in the fatigue test was very similar with the one with increasing load in the static test. The strain distribution could also be divided into three segments: the fully debonded segment, the stress transfer segment and the low stress segment. The fully debonded segment, started from loaded end of CFRP plate, would not be found during the early stage of fatigue tests but then formed and extended after certain cycles.

Typically, at the first cycle, the strain on the FRP plate decreased very quickly. This is because the FRP–concrete interface was intact, the stress on the FRP plate can transfer into the concrete through the interface within a relatively short distance. The lengths of this stress transfer segments in fatigue tests were slightly shorter than the ones obtained from static tests. This difference could be mainly attributed to the much lower load applied in fatigue tests, therefore a full length of the effective bond region [38] cannot be established.

Then with the increasing cycles, the strain on the FRP close to the load end would keep a higher level, which implied the debonding of the FRP plate. FT-4 as an example, the four curves show the strain profile on the FRP plate at the 1st, 1000th, 2000th and 5000th cycles, respectively. The first curve (black) expresses a sharp decreasing of the FRP strain, which implies no debonding occurred. The second curve (red) shows a short platform, which implies no stress transfer between FRP and concrete within this length. Combined with direct observation of the specimen, the length of this platform could be used to determine the debond length. As cycle numbers increased, the platform kept growing, which implies the growing of the debond length. However, the platform shows a downward trend at the 2000th (blue) and 5000th (pink) cycles. This is resulted from the friction between the debonded FRP plate and concrete, the friction would become more obvious as the length of debond increases.

By using this method, the debond length of each specimen at each cycle could be determined. The debond length and the debonding growth rate will be discussed in the next section.

#### 4.2.5. Debonding Growth Rate and a New Prediction Model

**Figure 12:**
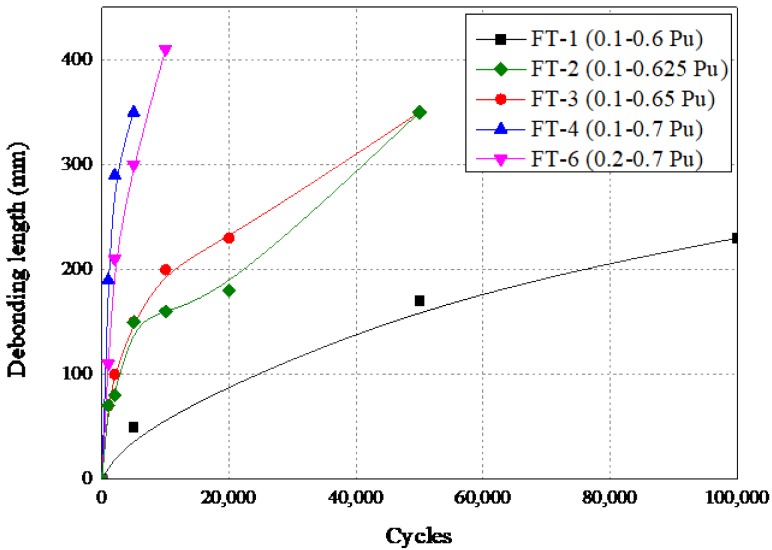
Debond lengths of each specimen at different cycles.

From analyzing the strain profile in Figure 11, also combined with direct observation during the tests, debond lengths at different cycles were determined. Furthermore, debonding growth rates were also investigated. The debond lengths of each specimen at different cycles were summarized in Figure 12. To be noted, the debond length measured by strain analyzing and observation were both taken at certain check cycles, so that debond length data may not be recorded when approaching fatigue failure of the bond joint. In other words, research in this paper took more concern into the initiation and propagation of the debonding growth rather than the final failure stage.

As shown in Figure 12, specimen with higher *P*_max_ led to more rapid debonding growth at the first stage, after which, debonding growth apparently slowed down. Debonding growth rate *da/dN* was defined as the entire debond length (*a*) at the last check cycle over total fatigue cycles (*N*) before the last check [35]. An increasing but still limited amount of studies have been conducted to establish the prediction model of the debonding growth rate of the FRP–concrete interface under fatigue loading. The majority of the prediction models were based on Paris law [41], which related *da/dN* with the range of stress intensity Δ*K* (corresponding to stress range) at the tip of a crack. The basic expression of the Paris law was shown below:

(1) $$\frac{da}{dN}=m{\left(\Delta K\right)}^{n}$$

where *m* and *n* are constants which can be obtained via experiments.

Diab et al. [35] presents an analytical solution of debond growth rate along the FRP–concrete interface and related it with the fracture energy ratio, $G_{\max}/G_{c}$. In addition, the fatigue–debond growth coefficient, *β*, was taken into account, which intended to involve the influence of the increasing debond length on the debond rate. The effect of fatigue loading frequency was also considered in the study. The final equation of debonding growth rate was given by:

(2) $$\frac{da}{dN}=m_{1}{\left(\frac{G_{\max}}{G_{c}}\right)}^{n_{1}}\cdot \beta$$

where *m*_1_ and *n*_1_ were parameters obtained from the linear regression of the experimental results.

Carloni et al. [36] provided an improvement of the equation above which explicitly took into account the amplitude and mean value of the fatigue load. However, the experimental results obtained in that study were not sufficient to characterize the coefficients. The improved equation was as follows:

(3) $$\frac{da}{dN}={\bar{m}}_{1}{\left(\frac{\alpha \sqrt{\Delta P\cdot \bar{P}}}{P_{crit}}\right)}^{{\bar{n}}_{1}}\cdot \bar{\beta }$$

where ΔP and $\bar{P}$ were the amplitude and mean value of the fatigue load range, respectively. $P_{crit}$ was the monotonic load–carrying capacity of the interface; α and $\bar{\beta }$ were the coefficient of the load frequency and the existing debond length; ${\bar{m}}_{1}$ and ${\bar{n}}_{1}$ were also parameters obtained from the linear regression of the experimental results. 

In order to reveal the relationship between the debonding growth rate and determinant parameters in fatigue tests of a certain FRP–concrete interface, a new prediction model was proposed in this paper. This new model was also based on Paris Law [41] and took the relative load amplitude, ΔS, and relative load level, $\bar{S}$, as the main characters, which could fully describe the fatigue loading profile. Considering all the fatigue tests in this work were taken at a frequency of 5 Hz, the influence of load frequency was not discussed. The new prediction model can be expressed as:

(4) $$\frac{da}{dN}=m{\left(\bar{S}\cdot \Delta S\right)}^{n}$$

(5) $$\bar{S}=\frac{P_{\max}-P_{\min}}{2P_{u}}$$

(6) $$\Delta S=\frac{P_{\max}+P_{\min}}{2P_{u}}$$

where $\bar{S}$ is the relative load level of the bond joint under fatigue loading; ΔS is the corresponding relative load amplitude; $P_{u}$ is the load capacity of the bond joint under static load and *m* and *n* were parameters obtained via experiments.

**Figure 13:**
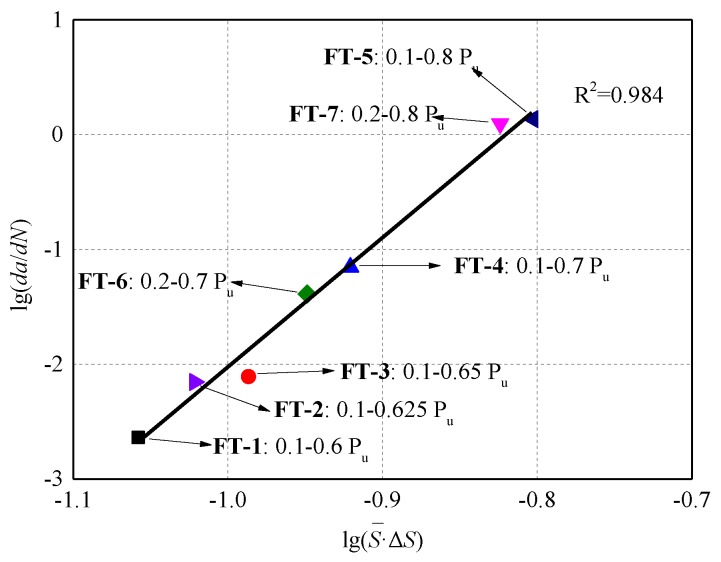
Logarithmic debonding growth rate, da/dN, versus the logarithmic product of the relative load level and relative load amplitude, $\left(\bar{S}\cdot \Delta S\right)$, of the experimental results.

Figure 13 shows the relationship between the logarithmic debonding growth rate,da/dN, and the logarithmic product of the stress level and stress amplitude, $\bar{S}\cdot \Delta S$, of the experimental results. Fitting the experimental results with a linear regression curve turns out the values of parameters *m* and *n* equaling 1 × 10^9.3^ and 11.3276, respectively. The equation fits very closely to the experimental results.

It should be noted that, referring to previous research, the parameters probably are affected by the lower limit of fatigue loading [35]. The dots in Figure 13, which present the experimental results of the fatigue tests in this work, include specimens with not only different upper fatigue loading limits, but also different lower limits. FT-6 and FT-7 were cyclically loaded under a lower limit as 0.2 *P*_u_, and the upper limit were respectively the same as FT-4 and FT-5, which were 0.7 *P*_u_ and 0.8 *P*_u_. As shown in Figure 13, the proposed equation can fit very well with specimens under fatigue loads with same load range but different load levels. Comparing FT-6 and FT-4, FT-7 and FT-5 turned out that higher lower limit could lead to slightly slower debonding propagation along the interface. Moreover, the debonding growth rate can be seriously influenced by the load level when the interface loaded with the same load range. FT-1 and FT-6, FT-4 and FT-7 were specimens respectively with the same load ranges but different load levels. Obviously, the load level plays a significant role in determining the debond growth rate, higher load levels cause rapider debonding growth, which correspondingly result in lower fatigue life of the interface. Furthermore, load range is still one of the key parameters that determine the debonding growth rate of the FRP–concrete interface. FT-6 and FT-5 shared the same load level, but FT-5 were loading with a greater load range. Same as steel rebar, when loaded with greater load range, the FRP–concrete interface endures a much more rapid debonding growth process and much shorter life span as well. 

## 5. Conclusions

In this paper, the main aim was to investigate the debond initiating and propagating process along the FRP–concrete interface under fatigue load. In order to analyze the debond length, the strain profile on the CFRP plate was recorded throughout the whole loading process at different check cycles. Moreover, visual observation was also adopted to define the actual debonding growth situation. Furthermore, a new prediction model of the debonding growth rate was proposed and had excellent agreement with the experimental results obtained from the fatigue tests in this work. Several conclusions can be obtained as follow:

(1) The failure modes of the FRP–concrete interface were the same whether under monotonic or fatigue load, both of which were debonding of CFRP plate with a thin layer of concrete attached on it;
(2) The proposed prediction model relates the fatigue debonding growth rate in FRP–concrete interface to the parameters, which can be easily obtained before the fatigue tests, such as the relative load range and load level, and shows an excellent agreement with the experimental results;
(3) The debond growth rate could be mainly affected by the fatigue load range and fatigue load level;
(4) A greater fatigue load range and higher fatigue load level could lead to a more rapid debond growth rate and lower fatigue life of the bond joint;
(5) Friction between the debonded FRP and the concrete substrate could play an important role in the fatigue behavior of the interface as debond propagating;
(6) Further studies are needed to reveal the influence of FRP stiffness, load frequency, adhesive properties and other possible factors.

## Figures and Tables

**Table 1:** Experimental program of static and fatigue tests.

Loading Type	Specimen	Load Range			
		$S_{\min}$	$S_{\max}$	ΔS	$\bar{S}$
Static	MT-1	Monotonically loaded to failure			
	MT-2				
	MT-3				
Fatigue	FT-1	0.1	0.6	0.25	0.35
	FT-2	0.1	0.625	0.2625	0.3625
	FT-3	0.1	0.65	0.275	0.375
	FT-4	0.1	0.7	0.3	0.4
	FT-5	0.1	0.8	0.35	0.45
	FT-6	0.2	0.7	0.25	0.45
	FT-7	0.2	0.8	0.3	0.5

**Table 2:** Physical properties of the adhesive.

Properties	Value/Description
Density	1.6 kg/L
Character	Grey viscous gel
Shear strength (steel to steel)	18.4 MPa

**Table 3:** Failure loads and cycles of monotonical and fatigue tests.

Specimen	$\begin{array}{l} P_{u}\\ \left(\mathrm{kN}\right) \end{array}$	$S_{\min}$	$\begin{array}{l} P_{\min}\\ \left(\mathrm{kN}\right) \end{array}$	$S_{\max}$	$\begin{array}{l} P_{\max}\\ \left(\mathrm{kN}\right) \end{array}$	ΔS	$\bar{S}$	$N_{f}$
MT-1	44.9	static tests						
MT-2	45.5							
MT-3	45.8							
FT-1	–	0.1	4.5	0.6	26.9	0.25	0.35	190,534
FT-2		0.1	4.5	0.625	28.1	0.2625	0.3625	65,317
FT-3		0.1	4.5	0.65	29.3	0.275	0.375	65,611
FT-4		0.1	4.5	0.7	31.5	0.3	0.4	9443
FT-5		0.1	4.5	0.8	36.5	0.35	0.45	332
FT-6		0.2	9	0.7	31.6	0.25	0.45	13,417
FT-7		0.2	9	0.8	36.0	0.3	0.5	227

Note: $N_{f}$ is the fatigue life of the specimen under fatigue loading.
